# Shape Memory Polymers Containing Higher Acrylate Content Display Increased Endothelial Cell Attachment

**DOI:** 10.3390/polym9110572

**Published:** 2017-11-03

**Authors:** Tina Govindarajan, Robin Shandas

**Affiliations:** Department of Bioengineering, University of Colorado at Denver|Anschutz Medical Campus, Aurora, CO 80045, USA; tina.govindarajan@ucdenver.edu

**Keywords:** shape memory polymer, acrylate, cardiovascular, stents, surface properties, endothelial cells

## Abstract

Shape Memory Polymers (SMPs) are smart materials that can recall their shape upon the application of a stimulus, which makes them appealing materials for a variety of applications, especially in biomedical devices. Most prior SMP research has focused on tuning bulk properties; studying surface effects of SMPs may extend the use of these materials to blood-contacting applications, such as cardiovascular stents, where surfaces that support rapid endothelialization have been correlated to stent success. Here, we evaluate endothelial attachment onto the surfaces of a family of SMPs previously developed in our group that have shown promise for biomedical devices. Nine SMP formulations containing varying amounts of tert-Butyl acrylate (tBA) and Poly(ethylene glycol) dimethacrylate (PEGDMA) were analyzed for endothelial cell attachment. Dynamic mechanical analysis (DMA), contact angle studies, and atomic force microscopy (AFM) were used to verify bulk and surface properties of the SMPs. Human umbilical vein endothelial cell (HUVEC) attachment and viability was verified using fluorescent methods. Endothelial cells preferentially attached to SMPs with higher tBA content, which have rougher, more hydrophobic surfaces. HUVECs also displayed an increased metabolic activity on these high tBA SMPs over the course of the study. This class of SMPs may be promising candidates for next generation blood-contacting devices.

## 1. Introduction

Cardiovascular disease, including atherosclerosis and related diseases, is one of the main causes of death worldwide [1]. Atherosclerosis is typically a result of a localized inflammatory response and can be characterized by plaque formation in blood vessels [1,2,3]. This plaque, which may consist of fat, cholesterol, calcium, blood components, etc., limits the blood flow through the blood vessel, potentially leading to an acute ischemic condition [4]. Traditionally, surgical intervention was used to open occluded vessels, but less invasive methods have become more prevalent [5]. Balloon angioplasty expanded occluded arteries, but re-obstruction of expanded vessels occurred relatively frequently, which required re-intervention at a later time [6,7].

Stents, or expandable tubes that are used to scaffold narrow and/or weakened arteries, provide support to blood vessels that have been re-opened, restoring the blood flow to organs and tissues downstream [8]. Stents have saved many lives, but limitations of current stents continue to drive research efforts to further improve their in vivo functionality [9]. Two primary limitations, restenosis, and thrombosis, arise from a lack of complete compatibility between the surface of the stent material and the surrounding physiological environment [1]. Efforts to tackle these limitations have focused on two areas: limiting local cell and tissue growth through drug elution; and, improving surface biocompatibility of the stent material.

Stent materials have a greater chance of survival in vivo if endothelialization of the device occurs soon after device implantation [10]. Rapid endothelialization is often characterized as significant cell presence within 24 h of implantation, with full cellular confluence achieved after three to seven days [11]. Although drug eluting stents (DES) have decreased the incidence of restenosis, they typically do not achieve rapid endothelialization, which may limit their long term utility [6,12,13,14]. As such, focus has turned to modifying the surface characteristics of stents to promote natural endothelialization. A variety of surface modification techniques, including physical, chemical, and biological methods have been evaluated on stent materials, including metals and polymers [13,15,16,17,18,19].

Endothelialization of materials for the successful integration of implanted biomedical devices was studied as early as the 1970s [20,21]. Endothelialization may occur by binding circulating endothelial progenitor cells or through endothelial cell migration from adjoining endothelium [10,22]. Once endothelial cells attach, usually within the first 24 h after device implantation, healthy cells proliferate, forming and retaining a permanent endothelial barrier on the surface of the device, resulting in reduced risk of long-term device rejection. Thus, if surface characteristics of the device allow for quick recruitment and proliferation of the endothelial lining after implantation, the chances of post-implant problems should decrease [23]. Rapid endothelialization also increases hemocompatibility, another imperative for successful integration of a cardiovascular stent [24]. The potential for surface modification to enhance biocompatibility has led to an increased interest in endothelialization studies, particularly for cardiovascular implants [23].

Shape memory polymers (SMPs) are one class of materials that are being considered for use in implanted, blood-contacting devices [25]. SMPs are smart materials that recover their original shape upon the application of an external stimulus [26,27,28,29,30,31,32,33]. These smart plastics are initially fabricated into their permanent shape and are then deformed and fixed into a temporary shape. These materials recover their original shape upon exposure to a stimulus such as heat, light, humidity, electrical, or magnetic fields, among others [28,31,32,34,35]. Their ability to recover from large deformations makes SMPs appealing as materials for biomedical devices, since such recovery allows for the implantation of these devices using minimally invasive techniques. 

Progress in SMP research is not limited to biomedical applications. Developments in information carriers for one-time identification, aerospace applications, smart textiles, polymer actuators, and sensors, active assembly/disassembly are also notable applications of SMPs [36,37,38,39,40,41,42]. High and low temperature SMPs are being developed for extreme environments, such as jet propulsion and aerospace applications [43,44]. Smart textile applications using SMPs range from aesthetic improvements, such as appeal, color changing capabilities, and soft display to functional applications, such as comfort, controlled drug release, wound monitoring, emotion sensing, extreme environment protection, etc. [37]. SMP actuators may be employed in adjustable rotation rate heat engines or self-regulating sun protectors for buildings [39]. Active assembly and disassembly should simplify and automate the processing procedures, resulting in high speed, low-cost disassembly, rendering parts useful for additional life cycles [41]. 

Since the shape memory effect, which drives the shape memory capabilities in SMPs, is a result of polymer structure and processing [24,26], prior work in biomaterials has focused on tuning bulk properties to meet the requirements of various medical applications [27,29,30]. Previous work from our group has focused primarily on thermomechanical properties, such as shape recovery, the shape memory effect (SME), and modulus, as well as cytotoxicity, but the surface compatibility of these acrylate-based SMPs has not been investigated [25,29,45,46]. In addition, there have been very few studies evaluating the surface characteristics of SMPs in the context of endothelial growth [34,35]. 

The purpose of this study was to examine the relationship between polymer characteristics of a well-studied acrylate-based SMP and endothelial cell attachment and growth. This would represent the first step in evaluating the potential for these materials for blood-contacting devices. Various compositions of SMP containing different weight percent ratios of tert-butyl acrylate (tBA) and poly(ethylene glycol) dimethacrylate (PEGDMA) were tested for endothelial cell attachment in vitro. The rapid endothelialization target for this study was high live cell presence after 24 h and complete cell sheet formation 72 h after cell seeding. 

## 2. Materials and Methods

### 2.1. Synthesis

**Figure 1:**
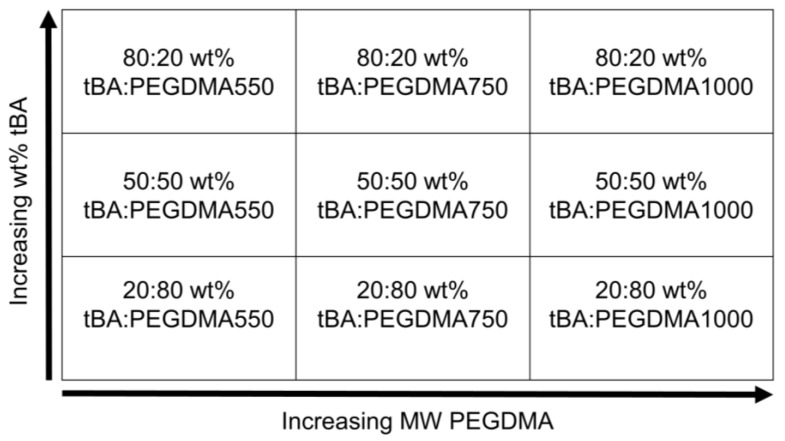
Shape Memory Polymers (SMP) formulation matrix of the nine formulations used.

Shape memory polymers were formulated using tert-butyl acrylate (tBA) and poly(ethylene glycol) dimethacrylate (PEGDMA), with average molecular weights (M_n_) of 550, 750, and 1000 with polymerization being facilitated by photoinitiator 2,2—dimethoxy-2-phenylacetophenone (DMPA). All of the products were obtained from Sigma-Aldrich (St. Louis, MO, USA), except for PEGDMA1000, which was obtained from Polysciences (Warrington, PA, USA) and were used as received. A total of nine polymer solutions were prepared from these monomer components (Figure 1).

Monomer mixtures were injected into molds composed of standard microscope slides (Thermo Fisher Scientific, Waltham, MA, USA), separated by a 1.33 mm silicone spacer (Mcmaster-Carr, Elmhurst, IL, USA), and cured under ultraviolet (UV) radiation of wavelength = 365 nm for 20 min, similar to previous methods [29]. The samples were then removed from the molds and post-cured in an oven at 75 °C overnight, similar to methods done previously [25].

### 2.2. Polymerization

Samples were post-processed at 75 °C in an oven overnight prior to use in characterization or cell attachment studies. Post-processing steps including annealing, which generated consistent physical properties and reduced material defects. Complete conversion of monomers was verified using Fourier transform infrared spectroscopy (FTIR, Nicolet Thermo Fisher Scientific, Waltham, MA, USA), as has been done previously [45]. FTIR samples were fabricated under similar conditions to those described above, but were made thinner, approximately 0.005 mm, to allow for the IR signal to penetrate the sample for FTIR analysis.

### 2.3. Dynamic Mechanical Analysis

Dynamic Mechanical Analysis (DMA) was performed using a TA Q800 DMA (TA Instruments, New Castle, DE, USA) and was used to verify the glass transition temperature (*T*_g_) of the various SMP formulations [47,48]. All of the samples were cut into specimens with dimensions of 20 mm × 5 mm × 1 mm for testing. Each sample was equilibrated to 0 °C for 1 min and heated to 100 °C at a rate of 3 °C/min. Testing was conducted at a frequency of 1.0 Hz and cyclic strain control at 0.1% strain.

### 2.4. Contact Angle

A Ramé-Hart goniometer (Ramé-Hart, Succasunna, NJ, USA) was used to obtain contact angle measurements and wettability of each SMP sample [49]. The wettability of each formulation was measured by applying water droplets to each surface and measuring the angle that formed between the water droplet and the surface of the sample. Measurements were taken 10 s after the 5 μL water droplet was introduced to the surface of the SMP to maintain consistency. Contact angles were measured using DROPImage Advanced computer software (Ramé-Hart, Succasunna, NJ, USA). Three different samples were analyzed per SMP formulation. Five drops were applied to each SMP sample surface and at least five measurements were taken per drop.

### 2.5. Atomic Force Microscopy (AFM)

Surface topography, a measure of the surface roughness of each SMP formulation, was obtained using atomic force microscopy (AFM) [50]. SMP fabrication molds were made using new glass microscope slides, as done previously, which were cleaned using detergent and diH_2_O, followed by ethanol and acetone, and a final rinse using diH_2_O to remove any surface artifacts on the glass. These measures were taken to ensure that the surface features detected by the AFM were a result of the changes in weight percent or molecular weight of the PEGDMA. Images were obtained using a NanoSurf easyScan 2 (Nanomaterials Characterization Facility, University of Colorado, Boulder, CO, USA). Image post-processing was completed using Gwyddion open source software (Gwyddion, Brno, Czech Republic). The root mean square roughness coefficient, *R*_q_, measured by the standard deviation of the distribution of surface heights, also obtained from Gwyddion, provided quantitative information of the sample surface [51]. 

### 2.6. Cell Culture

Human umbilical vein endothelial cells (HUVECs), obtained from the endothelium of the umbilical vein, are a common cell model for angiogenesis and re-endothelialization studies. HUVECs are also robust, making them a favorable cell type for use in such studies, and as a result, HUVECs were the chosen cell model for this re-endothelialization study [23].

Prior to cell culture experiments, human umbilical vein endothelial cells (HUVECS) (Lonza, Walkersville, MD, USA) were seeded in T-75 flasks using complete growth medium: EBM-2 Cell Culture Bullet Kit (Lonza, Walkersville, MD, USA). HUVECs were maintained in conditions of 37 °C and 5% CO_2_ in a humidified incubator. Cells were washed with HEPES, 1M, buffer (Life Technologies, Carlsbad, CA, USA) prior to the changing of the media. Media was changed every two to three days, and cells were passaged at 80–90% confluence. Cell passages two through six were used for cell seeding on SMP substrates. All of the experiments were conducted in triplicate.

### 2.7. Light Microscopy

To monitor general cell health and ensure that there were no signs of contamination present, HUVECs and HUVEC-SMP samples were observed under an inverted light microscope (Nikon, Melville, NY, USA) daily.

### 2.8. Live/Dead Assay

SMP samples were submerged in growth media and were equilibrated to 37 °C for 24 h prior to cell seeding. HUVECs were then plated on 1cm diameter SMP substrates in 24-well plates and were allowed to attach. Cells were seeded at a seeding density of 1 × 10^5^ cells/mL per well. Daily monitoring of cell-adherent SMPs using transmission microscopy allowed for the qualitative assessment of proper cell growth and the absence of contamination. Cell viability was quantitatively assessed at two time points, approximately 24 h after plating and again at approximately 72 h. Complete cell medium was changed daily to ensure that cells received consistent nourishment during the study. The Live/Dead Cell Imaging Kit (488/570) (Life Technologies, Carlsbad, CA, USA) was used to assess endothelial cell attachment and viability through fluorescent staining. Live cells, which were actively attached to the substrate, emit green fluorescence, while dead cells fluoresce red. Images were obtained using an EVOS FL Cell Imaging System (Life Technologies, Carlsbad, CA, USA). At least three images from three replicate experiments were used for cell attachment counting using ImageJ software (NIH).

### 2.9. Cell Metabolism

PrestoBlue^®^ Cell Viability Reagent (Life Technologies, Carlsbad, CA, USA), a plate-based resazurin assay, was added to cell-substrate samples and left to incubate for 2 h. This cell viability reagent, when added to cells, exploits the reducing power of cells to quantitatively measure cell metabolism. This also provides an indirect measurement of cell proliferation and cytotoxicity [52]. Media was removed from samples after the two-hour incubation period and fluorescence was measured on a Synergy 2 microplate reader (BioTek, Winooski, VT, USA).

### 2.10. Statistical Analysis

The data were expressed as mean ± standard deviation. Statistical analysis was performed using MATLAB (MathWorks, Natick, MA, USA), and significance was determined using ANOVA (with α-level of significance set to 0.05). The Tukey’s Honest Significant Difference Test assessed the significance between individual samples if ANOVA determined significance of the sample set. 

## 3. Results

### 3.1. Dynamic Mechanical Analysis

**Figure 2:**
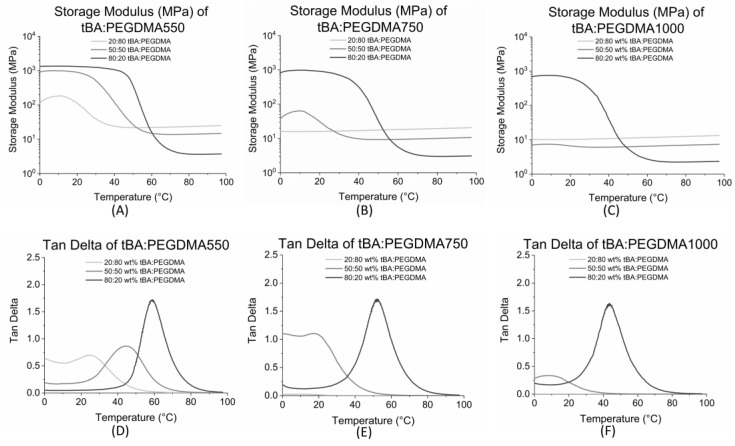
(**A**) Storage Modulus (MPa) of tert-Butyl acrylate (tBA): Poly(ethylene glycol) dimethacrylate (PEGDMA)550; (**B**) Storage Modulus (MPa) of tBA:PEGDMA750; (**C**) Storage Modulus (MPa) of tBA:PEGDMA1000. Glassy regions are absent for the formulations containing 50 wt % tBA or less; (**D**) Tan delta of tBA:PEGDMA550; (**E**) Tan delta of tBA:PEGDMA750; (**F**) Tan delta of tBA:PEGDMA1000. *T*_g_ increases with increasing tBA content and decreases with increasing PEGDMA MW.

Select bulk properties of SMP formulations were characterized using storage modulus and tan delta (δ) curve data, obtained from dynamic mechanical analysis (DMA) [47]. The plateaus above and below *T*_g_ on the storage modulus curves represent the glassy and rubbery moduli, respectively. As shown in Figure 2A–C, the glassy regions are absent for most formulations containing 50 weight percent tBA or less. Rubbery modulus increases with an increasing PEGDMA content, as shown previously [25].

The peak of the tan delta curve was used to determine the activation temperature or glass transition temperature, *T*_g_, which is the temperature at which the material can revert from its temporary shape back to its permanent shape. The onset of shape recovery, *T*_onset_, which is the beginning of the shape recovery transition, as well as the *T*_g_ range, was calculated using the methods described previously and are displayed in Table 1 [25]. DMA data for some of the samples has been analyzed by our group in previous experiments; our data agreed with prior results [25,29,48,53].

Increased monomer (tBA) content resulted in increased *T*_g_, so

(*T*_g_ 80:20 wt % tBA:PEGDMA) > (*T*_g_ 50:50 wt % tBA:PEGDMA) > (*T*_g_ 20:80 wt % tBA:PEGDMA)

for a given crosslinker.

Decreasing the molecular weight of the PEG component in PEGDMA also increased *T*_g_, which indicates that the samples containing PEGDMA550 had a higher *T*_g_ than samples containing PEGDMA750, which in turn had higher *T*_g_ than samples containing PEGDMA1000, for a given weight percent ratio (Figure 2D–F).

### 3.2. Contact Angle

**Figure 3:**
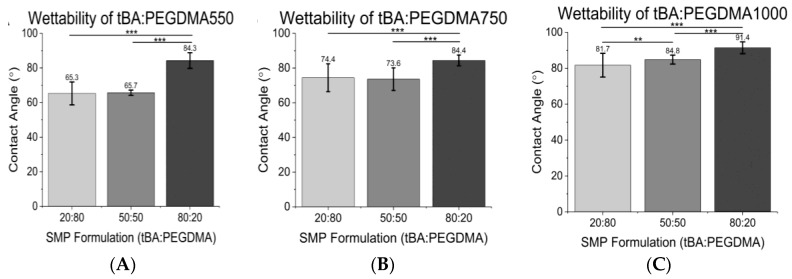
(**A**) Wettability of tBA:PEGDMA550; (**B**) Wettability of tBA:PEGDMA750; (**C**) Wettability of tBA:PEGDMA1000. Wettability decreases (increasing hydrophobicity) with increasing tBA content and increasing crosslinker (PEGDMA) MW. Significance was determined using one-way ANOVA to determine significant differences between samples of a given crosslinker in addition to the Tukey’s Honest Significance Difference Procedure between individual samples. * corresponds to *p* < 0.05, ** corresponds to *p* < 0.01, *** corresponds to *p* < 0.001.

Contact angle increased with increasing tBA content for a given crosslinker (Figure 3A–C). Thus,

(CA 80:20 wt % tBA:PEGDMA) > (CA 50:50 wt % tBA:PEGDMA) > (CA 20:80 wt % tBA:PEGDMA formulations).
(1)

Specifically, water contact angles increased 11–23% from the 20:80 wt % tBA:PEGDMA formulations to the 80:20 wt % tBA:PEGDMA formulations and 7–22% between the 50:50 wt % tBA:PEGDMA and the 80:20 wt % tBA:PEGDMA groups. Additionally, the wettability decreased with increasing crosslinker length for a given weight percent of crosslinker, i.e., samples containing PEGDMA1000 were more hydrophobic than those containing PEGDMA550.

### 3.3. Atomic Force Microscopy (AFM)

**Figure 4:**
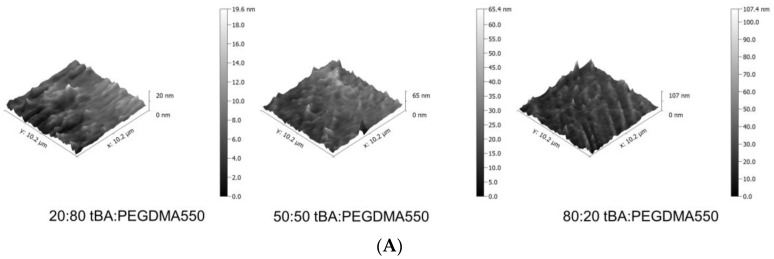

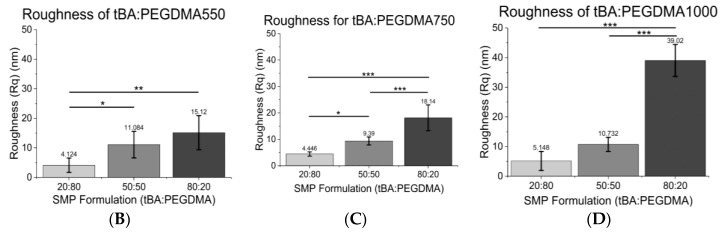
(**A**) Representative Atomic Force Microscopy (AFM) images of tBA:PEGDMA550 samples. 3D AFM images depict increases in surface roughness as tBA increases. tBA:PEGDMA750 and tBA:PEGDMA1000 AFM images follow a similar trend; (**B**) Root mean square roughness (*R*_q_) of tBA:PEGDMA550; (**C**) Root mean square roughness (*R*_q_) of tBA:PEGDMA750; (**D**) Root mean square roughness (*R*_q_) of tBA:PEGDMA1000. Root mean square roughness (*R*_q_) generally increases with increasing tBA content and increasing PEGDMA MW. Significance was determined using one-way ANOVA to determine significant differences between samples of a given crosslinker in addition to the Tukey’s Honest Significance Difference Procedure between individual samples. * corresponds to *p* < 0.05, ** corresponds to *p* < 0.01, *** corresponds to *p* < 0.001.

AFM imaging was used to assess the topographical features present on each SMP surface, quantified by using the root mean square surface coefficient, *R*_q_. As seen in Figure 4A–D, the roughness increased with increasing tBA content, so 80:20 wt % tBA:PEGDMA formulations were roughest while the 20:80 wt % tBA:PEGDMA formulations were smoothest for a given crosslinker. Roughness increased 73–95% between the 80:20 wt % tBA:PEGDMA group and the 20:80 wt % tBA:PEGDMA group and increased 23–68% from the 50:50 wt % tBA:PEGDMA formulation to the 80:20 wt % tBA:PEGDMA formulation. Additionally, samples containing PEGDMA1000 were rougher than those containing PEGDMA550 for a given weight percent ratio.

### 3.4. Cell Viability

**Figure 5:**
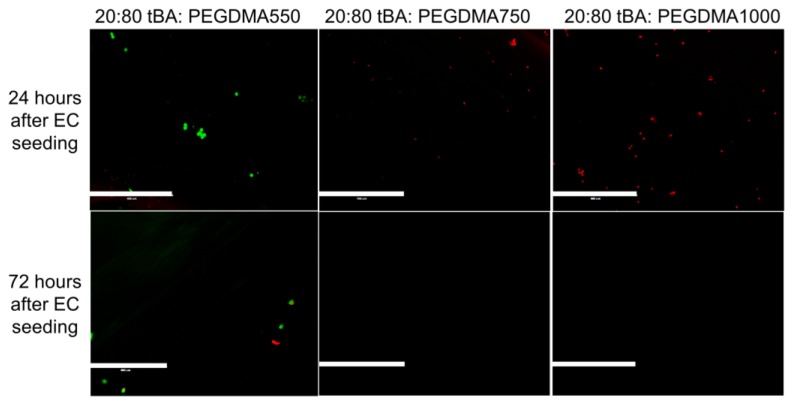
Live-Dead Analysis of SMP formulations with the lowest weight percent of monomer (20 wt % tBA). These samples show little to no endothelial cell attachment and have a high presence of dead endothelial cells. Scale bar = 400 µm.

Cell viability, characterized as endothelial cell attachment on top of the SMP substrate, was monitored using both light and fluorescence microscopy. Results for SMP formulations containing the lowest amount of tBA (20 weight percent) are shown in Figure 5. These samples displayed little or no live HUVEC presence 24 h after cell seeding, but the presence of dead cells was prevalent indicating that few cells survived after 72 h.

**Figure 6:**
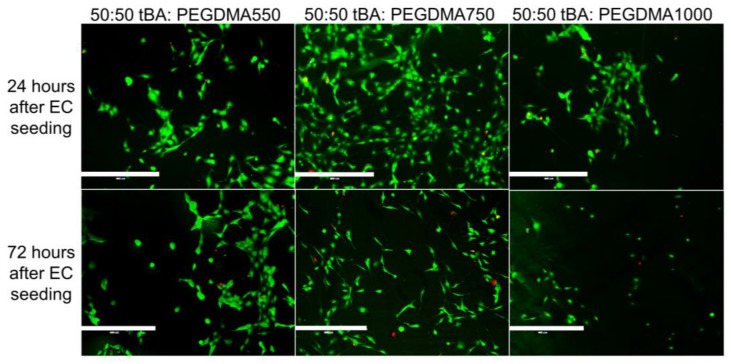
Live-Dead Analysis of SMP formulations with equal weight percent monomer (tBA) and crosslinker (PEGDMA). There are endothelial cells present on the surface of all samples regardless of crosslinker length, but there is some variability based on the crosslinker used in the sample. Specifically, both PEGDMA550 and PEGDMA750 samples seem to support more HUVEC attachment as compared to the PEGDMA1000 sample. Scale bar = 400 µm.

SMP formulations containing equal weight percent monomer and crosslinking agent, 50:50 wt % tBA:PEGDMA, displayed the greatest variability in endothelial cell viability (Figure 6). These formulations showed endothelial cell presence 24 h after HUVEC introduction, but viability and cell attachment decreased 72 h after cell introduction. 

**Figure 7:**
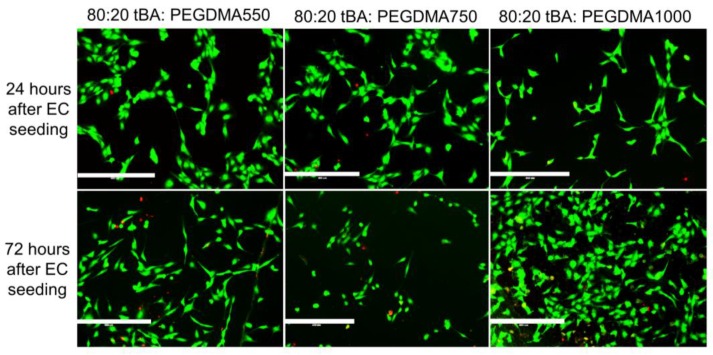
Live Dead Analysis of SMP formulations with highest weight percent (80 wt %) monomer (tBA). Endothelial cell attachment is indicated by the high number of living cells and the low number of dead cells present on the samples. Scale bar = 400 µm.

SMPs with the highest tBA content, 80 weight percent, showed the highest amount of endothelial cell attachment, displaying 4–89% greater endothelial cell presence 24 h after cell introduction and 33–100% increased cell presence after 72 h when compared to the other formulations. These samples also had the highest ratio of live cells to dead cells (Figure 7). 

The 80:20 wt % tBA:PEGDMA1000 sample initially displayed less endothelial cell attachment when compared to the other formulations with 80 weight percent monomer, but after 72 h, cell presence increased, an indication of healthy endothelial cells. The 80:20 wt % tBA:PEGDMA750 formulation supported cell attachment 24 h after HUVEC introduction, and was able to retain most cells after 72 h. The final sample, 80:20 wt % tBA:PEGDMA550, displayed HUVEC attachment 24 h after cell seeding, and was able to retain cell attachment 72 h after initial introduction. All of the samples containing 80:20 wt % tBA:PEGDMA had few dead cells present, if any.

**Figure 8:**
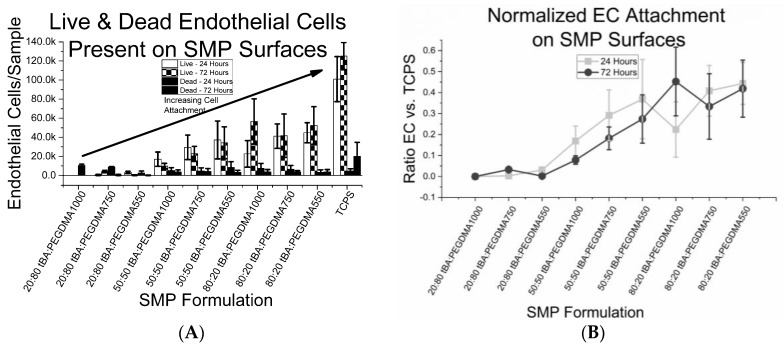
(**A**) cell count of human umbilical vein endothelial cell (HUVECs) present on each sample, scaled to size of the SMP sample. Living endothelial cells are present on sample containing at least 50 wt % tBA; (**B**) endothelial cell count of each SMP sample normalized to endothelial cell count of control sample (TCPS). While HUVECs attach to SMP surfaces, full coverage of SMP samples is yet to be achieved.

We found that EC attachment occurred on samples containing at least 50 weight percent tBA, as seen in Figure 8A. However, even though these formulations displayed endothelial cell attachment, the samples did not display cell sheet formation after 72 h. The largest ratio of EC coverage on a sample compared to the tissue culture plate control, which displayed a full endothelial cell sheet after 72 h, is approximately 0.4, as displayed in Figure 8B.

**Figure 9:**
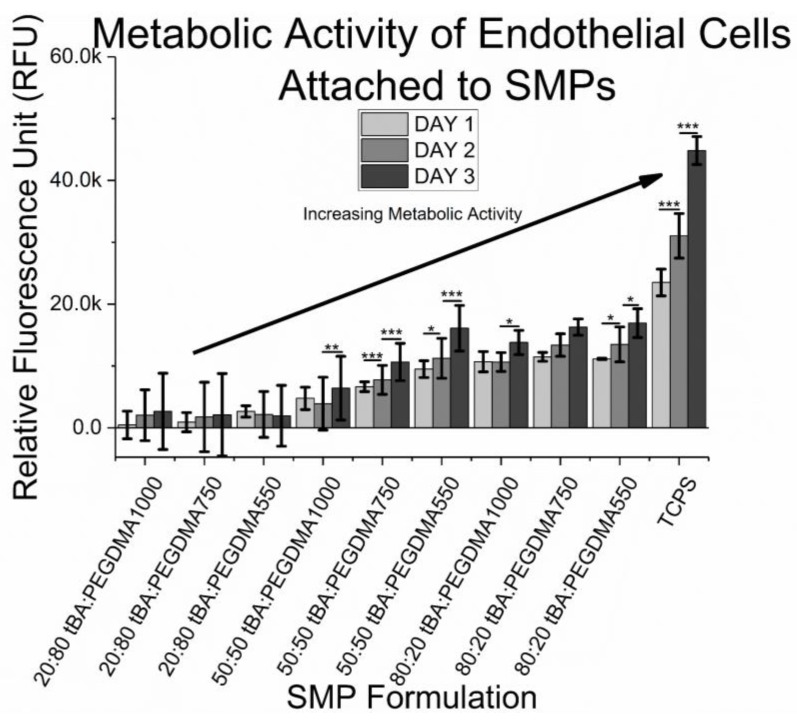
Cytocompatibility of SMPs. There is evidence of increasing metabolic activity, prominently 72 h after cell introduction, but some samples show evidence of metabolic activity increasing just 48 h after cell seeding. Samples that are cytotoxic have little metabolic activity as compared to samples that are cytocompatible, confirming a lack of HUVEC presence. Significance was determined using one-way ANOVA to determine significant differences between samples of a given wt % ratio and crosslinker combination, in addition to the Tukey’s Honest Significance Difference Procedure between individual samples. * corresponds to *p* < 0.05, ** corresponds to *p* < 0.01, *** corresponds to *p* < 0.001.

Cell metabolism was measured daily for 72 h, with results being displayed in Figure 9. Resazurin, which is initially non-fluorescent, is reduced to a fluorescent resorufin when added to healthy cells. Increases or decreases in reduction provide insight into cell health, such as metabolism and cytotoxicity [52]. Samples containing 20 wt % tBA did not show any signs of resazurin reduction, further confirming that if any cells were present on the samples, the cells were unhealthy, dying, or already dead. Most of the samples containing 50 wt % tBA and 80 wt % tBA showed signs of increasing resazurin reduction, which may be an indication of an increased endothelial cell presence, and consequently, possible cell proliferation. 

## 4. Discussion

Current stent technologies have found broad clinical utility, but continue to encounter issues, such as restenosis and/or thrombosis, both of which may require subsequent reintervention to prevent further complications. While there has been extensive work on fine-tuning the bulk mechanical properties of SMPs and some work on cytotoxicity and biocompatibility for applications such as hernia meshes, embolic coils, and stent grafts from our group as well as others, little work has focused on surfaces, and more specifically, the cytocompatibility and hemocompatibility of these materials [29,35,46,54,55].

Previous work from our group has addressed bulk mechanical properties, such as thermomechanical behavior, the shape memory effect, partially constrained and free recovery, biocompatibility, and cytotoxicity of these tBA:PEGDMA SMPs. However, previous studies have not reported on surface properties or endothelial cell attachment on the surface of these materials [25,29,45,46,48]. Endothelialization of implanted biomedical devices increases the likelihood of device integration due to improvements in hemocompatibilty and a reduced risk of device rejection, which necessitates optimization of surfaces to encourage HUVEC attachment [23,24,25]. 

This study evaluated the ability of select acrylate-based shape memory polymers to attach and retain endothelial cells. Numerous studies have shown that interactions between a material’s surface and its surroundings play a notable role in dictating the success of an implanted biomedical device [3,56,57]. While previous studies from our lab have assessed the bulk mechanical properties of acrylate-based SMPs for stent use, no studies have evaluated the surface characteristics of these SMPs in detail.

Our group has previously examined the activation temperature for the SMP formulations used in this study [25,29,48]. Briefly, the 80:20 wt % tBA:PEGDMA1000 and the 50:50 wt % tBA:PEGDMA550 samples have glass transition temperatures closest to body temperature, with *T*_g_’s close to 44 °C for both formulations. The remaining 80:20 wt % formulations had higher *T*_g_’s, but our group has shown that these formulations are still able to exhibit shape memory at physiological temperature [48]. Prior data has shown that varying the crosslinking agent between 10% and 40% does not significantly impact *T*_g_ for related crosslinking agents [25]. However, when crosslinker content exceeds 40% weight percent, the transition regime between glassy and rubbery becomes blurry and depletes shape memory ability, which may cause the larger differences in *T*_g_, as displayed here [58]. Additionally, glassy modulus and the transition between glassy to rubbery state is nearly non-existent for formulations containing 50 wt % or less, which also demonstrates the depletion of the shape memory ability. Increases in rubbery modulus and the reductions in stiffness are seen as PEGDMA content increases, agreeing with prior data [25]. All of the samples containing 20:80 wt % tBA:PEGDMA, as well as the 50:50 wt % tBA:PEGDMA750 and the 50:50 wt % tBA:PEGDMA1000 formulations have considerably lower *T*_g_’s and exhibit breakage when deformed at room temperature due to a higher rubbery modulus and reduced stiffness, and would thus not exhibit the shape memory effect at 37 °C. 

SMP formulations with higher *T*_g_’s are stiffer at physiological temperature. Specifically, the 20:80 wt % tBA:PEGDMA1000 sample, with a glass transition temperature of approximately 6 °C, exhibited a storage modulus of 11.27 MPa, whereas the 80:20 tBA:PEGDMA550 SMP, with a glass transition temperature of 60 °C, had a storage modulus of 1194 MPa. This confirms that the samples with *T*_g_’s lower than 37 °C are not as stiff as the samples with glass transition temperatures greater than 37 °C. The large difference in storage modulus between the two formulations provides additional insight into material properties at 37 °C, which could be an important factor in material choice. For a device that is going to serve as a support mechanism, a higher stiffness may be more desirable, as would be the case for a cardiovascular stent in a blood vessel. 

Numerous studies have cited substrate stiffness as an important factor in determining cell attachment to a substrate [59,60]. These studies found that stiffer samples often exhibit higher endothelial cell attachment when compared to their softer counterparts [55]. Our data affirm these prior results. Stiffer SMPs, or those with activation temperatures above 37 °C, displayed greater endothelial cell attachment and viability. Specifically, all of the 80:20 wt % tBA:PEGDMA formulations, as well as the 50:50 wt % tBA:PEGDMA550 sample, exhibited endothelial cell attachment and retained attached cells for up to 72 h. Thus, SMPs with a *T*_g_ that is slightly higher than body temperature, and therefore increased stiffness, appear well suited for endothelial cell attachment, similar to results found in other studies [59]. 

Contact angle measurements provided quantitative wettability data of each surface [49]. Surfaces exhibiting a moderate wettability have shown a higher affinity for cell attachment as compared to surfaces with extreme wettability [23]. Formulations with higher PEGDMA content are more hydrophilic, as are formulations with lower molecular weight PEG chains, i.e., samples containing PEGDMA550. PEGDMA is commonly used in hydrogels for its hydrophilic tendencies, as well as its highly tunable material properties, which explains the smaller contact angles for samples containing higher amounts of PEGDMA [61]. Since the formulations were based on weight percent ratios of tBA and PEGDMA, the greater hydrophilicity of samples containing PEGDMA550 could be the result of an increased PEG presence in the sample as compared to a sample containing PEGDMA1000. Despite these trends in wettability, the differences in wettability did not appear significant enough to have a pronounced effect on endothelial cell attachment to these surfaces. This is analogous to results found in other studies where changing PEG length produced large variations in cell attachment [62,63]. 

Surface roughness was also analyzed for each sample. Roughness increased with an increasing tBA content and with molecular weight. Rougher surfaces also had higher contact angles, and have been shown to be more conducive to cell attachment, which is consistent with other studies [64,65,66]. Even though neither surface roughness nor wettability appears to be a sole deciding factor of HUVEC attachment to the SMP surfaces, both aspects have been shown to affect endothelial cell attachment. It is often unclear which factor may play the dominant role in cell attachment, due to the complex nature of cell-surface interactions, as seen in other studies [67]. 

HUVEC attachment was assessed using fluorescence microscopy. Formulations containing the lowest weight percent monomer, 20:80 wt % tBA:PEGDMA, displayed very little cell attachment within 24 h, leading to minimal or no HUVEC presence 72 h after cell introduction. Since these formulations have high PEG content, and PEG has been shown to resist protein and cell attachment, the presence of dead cells or lack of cells appears reasonable [68]. However, recent studies have also indicated that some PEGDMA-based hydrogels may support the cell adhesion of certain cell types, requiring the consideration of these low tBA materials [69]. The absence of dead cells between 24 h and 72 h can be explained by the tendency of dead endothelial cells to detach because there is no active mechanism for dead cells to remain tethered to the surface of the SMP. The dead cells are then removed when the samples are washed with buffer or cell culture medium is replenished. 

Formulations containing equal amounts of tBA and PEGDMA displayed the greatest amount of variation in endothelial cell attachment. The 50:50 wt % tBA:PEGDMA1000 sample behaved more like the formulations containing 20 weight percent tBA, supporting little HUVEC attachment initially and showing a decrease in HUVEC presence after 72 h, indicating that this formulation may not support endothelial attachment. The 50:50 wt % tBA:PEGDMA750 and the 50:50 wt % tBA:PEGDMA550 samples behaved more similarly to formulations containing 80 weight percent tBA, displaying HUVEC attachment 24 h after cell seeding and retaining a small number of attached cells after 72 h.

The SMP formulations containing 80:20 wt % tBA:PEGDMA exhibited the greatest HUVEC attachment. These samples displayed cell attachment and retained endothelial cells 72 h after initial cell seeding. Some samples even showed indications of increased EC presence, which may indicate cell proliferation. Thus, these formulations may be good candidates for use in implanted devices that require rapid endothelialization to succeed, such as cardiovascular stents. Further work, including in vivo evaluation, would be required, to confirm that these formulations would be good candidates for stent fabrication. 

The SMP samples used in this study were solid surfaces, whereas stents are often perforated tubes, such as those that have been fabricated by our group in previous work [52]. Perforated SMP stents are easy and inexpensive to fabricate, unlike metals, and these perforated SMPs may experience greater endothelial cell surface coverage due to migration of ECs from adjoining healthy endothelium in addition to endothelial progenitor cell (EPC) attachment and should verified in subsequent studies. Due to the exploratory scope of this initial study, additional experiments evaluating the effect of surface roughness on endothelial cell attachment and viability were not included, but will be conducted in future studies. Protein adsorption to the sample surface from the culture medium has been shown to encourage cell attachment, and SMPs that displayed cell attachment may have demonstrated more selective protein adsorption from the cell culture medium, allowing for ECs to attach long enough to produce their own adhesion proteins, which should be verified in future work [9,67,70,71]. Finally, in vivo studies would be an important next step to confirm the in vitro results.

## 5. Conclusions

We investigated endothelial cell attachment and survival on the surface of select acrylate-based SMPs to use these materials for cardiovascular stent fabrication. SMP formulations containing a high weight percent monomer, 80:20 wt % tBA:PEGDMA, yielded the highest endothelial cell attachment 24 h after cell introduction. These formulations also exhibited low cytotoxicity, as shown by minimal cell death 72 h after cell seeding. The chemical composition of the SMP surface seems to have the greatest influence on both surface properties and cell attachment, as it affects the material properties that encourage or discourage endothelial cell attachment. Optimizing the SMP surface to encourage endothelial cell sheet formation is the next step towards implementing these materials in stent fabrication. 

## Figures and Tables

**Table 1:** *T*_g_, Tonset and *T*_g_ range for SMP Formulations.

Formulation	*T*_g_ (°C)	*T*_onset_ (°C)	*T*_g_ Range (°C)
20:80 tBA:PEGDMA1000	6 ± 2	-	-
20:80 tBA:PEGDMA750	11 ± 1	-	-
20:80 tBA:PEGDMA550	25 ± 1	15 ± 2	19 ± 5
50:50 tBA:PEGDMA1000	10 ± 1	8 ± 3	3 ± 5
50:50 tBA:PEGDMA750	19 ± 2	12 ± 1	13 ± 4
50:50 tBA:PEGDMA550	44 ± 1	25 ± 3	38 ± 7
80:20 tBA:PEGDMA1000	44 ± 1	26 ± 3	37 ± 4
80:20 tBA:PEGDMA750	52 ± 1	35 ± 1	32 ± 1
80:20 tBA:PEGDMA550	60 ± 3	47 ± 3	24 ± 2
